# Process parameter development for the scaled generation of stem cell‐derived pancreatic endocrine cells

**DOI:** 10.1002/sctm.21-0161

**Published:** 2021-08-13

**Authors:** Diepiriye G. Iworima, Sebastian Rieck, Timothy J. Kieffer

**Affiliations:** ^1^ School of Biomedical Engineering University of British Columbia Vancouver British Columbia Canada; ^2^ Department of Cellular and Physiological Sciences University of British Columbia Vancouver British Columbia Canada; ^3^ ViaCyte Inc San Diego California USA; ^4^ Department of Surgery University of British Columbia Vancouver British Columbia Canada

**Keywords:** cell culture, cell transplantation, clinical translation, diabetes, differentiation, pancreatic differentiation, pluripotent stem cells, stem cell culture

## Abstract

Diabetes is a debilitating disease characterized by high blood glucose levels. The global prevalence of this disease has been projected to reach 700 million adults by the year 2045. Type 1 diabetes represents about 10% of the reported cases of diabetes. Although islet transplantation can be a highly effective method to treat type 1 diabetes, its widespread application is limited by the paucity of cadaveric donor islets. The use of pluripotent stem cells as an unlimited cell source to generate insulin‐producing cells for implant is a promising alternative for treating diabetes. However, to be clinically relevant, it is necessary to manufacture these stem cell‐derived cells at sufficient scales. Significant advances have been made in differentiation protocols used to generate stem cell‐derived cells capable of reversing diabetes in animal models and for testing in clinical trials. We discuss the potential of both stem cell‐derived pancreatic progenitors and more matured insulin‐producing cells to treat diabetes. We discuss the need for rigorous bioprocess parameter optimization and identify some critical process parameters and strategies that may influence the critical quality attributes of the cells with the goal of facilitating scalable manufacturing of human pluripotent stem cell‐derived pancreatic endocrine cells.

## Graphical abstract

Donor islet transplantation can effectively reverse hyperglycemia in people living with type 1 diabetes. However, there is a limited supply of replacement donor islets. Human pluripotent stem cells are a renewable source of starting material that could overcome the supply challenges associated with islet transplantation. Pluripotent stem cells can be expanded at large scale, differentiated into islet‐like clusters, and cryopreserved prior to implant into patients.

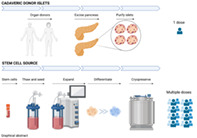

**Significance statement**

Diabetes is a global pandemic that can potentially be treated using stem cell‐derived pancreatic progenitors and hormone‐secreting cells. The ability to generate stem cell‐derived derivates at sufficient scales is a critical step toward using these cells to treat diabetes. This article reports on some critical process parameters and quality attributes that affect the final stem cell‐derived product.

## 1 INTRODUCTION

Diabetes is ranked within the top 10 most deadly diseases worldwide.^1^, ^2^ According to the World Health Organization, as of 2019, 463 million adults had diabetes, and that number is predicted to rise to 700 million by 2045.^3^ It is estimated that by 2030, accumulated global diabetes management expenditures will rise to $2.48 trillion.^4^ All forms of diabetes are characterized by chronically elevated blood glucose levels. Glucose homeostasis is regulated by the actions of alpha and beta cells contained within the islets of Langerhans in the pancreas. Alpha cells secrete more glucagon during periods of low blood glucose (hypoglycemia), while beta cells secrete higher levels of insulin during periods of elevated blood glucose (hyperglycemia). Type 1 diabetes (T1D) is an autoimmune disease caused by insulin insufficiency due to the destruction of insulin‐producing beta cells. T1D requires lifelong insulin therapy either by exogenous or endogenous sources.

**FIGURE 1:**
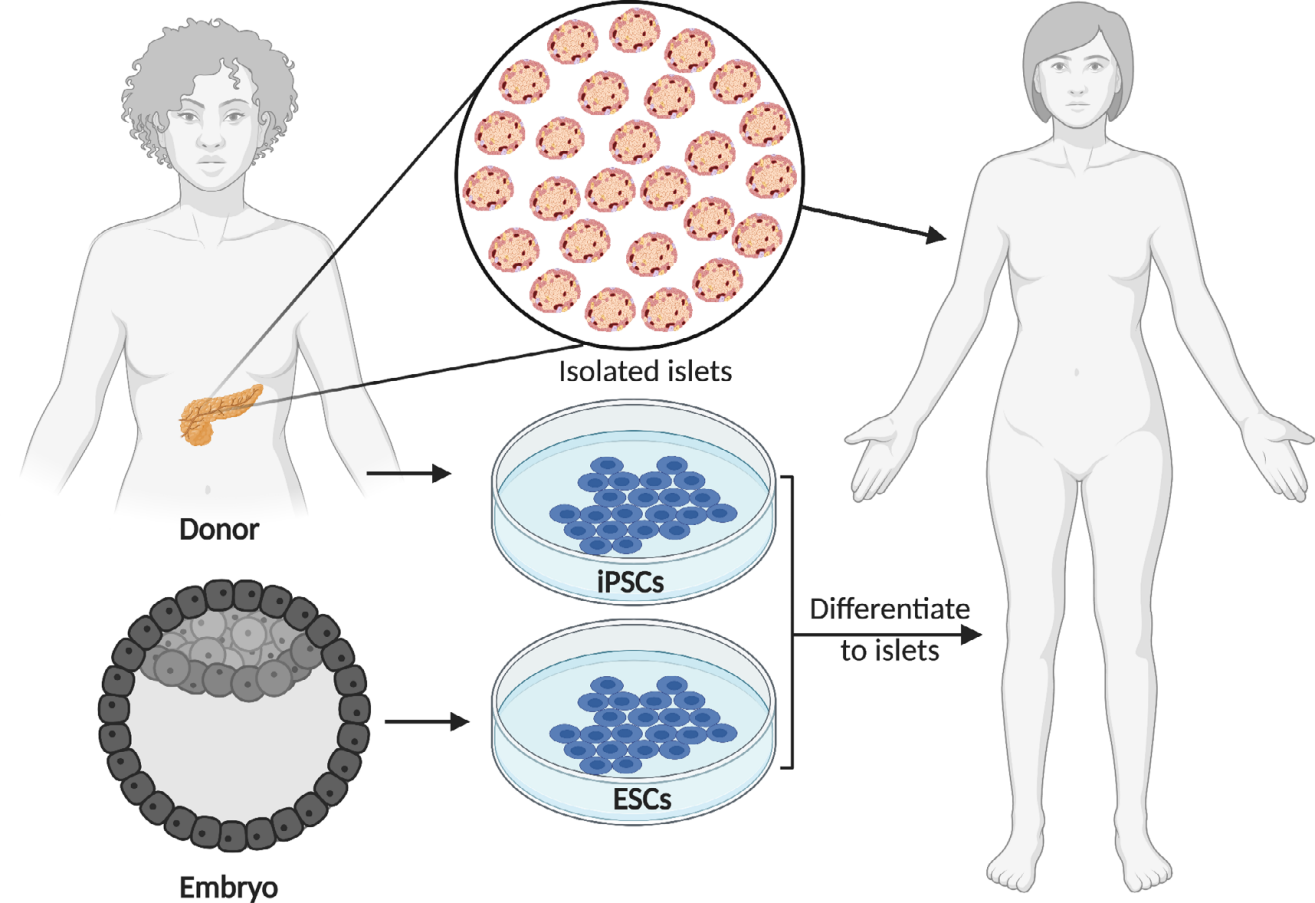
Schematic of potential cell sources of insulin‐producing cells that could be used for replacement therapy in diabetes. ESC, embryonic stem cell; iPSC, induced pluripotent stem cell

Although great strides have been made since the discovery and isolation of insulin by Drs. Banting, Best, Collip, and Macleod in 1922,^5^ patients with T1D still experience compromised quality of life. The most common method for managing blood glucose levels is administering multiple daily insulin injections. However, improper dosing of insulin puts patients at risk of life‐threatening hypoglycemia and a myriad of long‐term complications resulting from prolonged hyperglycemia. Also, insulin injections cannot mimic the precise glycemic control of pancreatic islets. The most effective therapy for reversing hyperglycemia in T1D is the transplantation of islets isolated after brain or cardiac death.^6^, ^7^, ^8^ Islet transplantation demonstrates the efficacy of beta cell replacement for the treatment of T1D. However, the scarcity of donors necessitates the need for other sources of insulin‐producing cells in order to support widespread adoption of this approach (Figure 1).

Pluripotent stem cells (PSCs) include embryonic stem cells (ESCs) and induced pluripotent stem cells (iPSCs). ESCs are derived from the inner cell mass of a blastocyst, whereas iPSCs are generated by reprogramming somatic cells to a stem cell state. Both ESCs and iPSCs can theoretically proliferate indefinitely and can differentiate into cells from all three germs layers. The use of PSC‐derived insulin‐producing cells as an alternative therapy for the treatment of T1D can address the problem of donor islet scarcity associated with islet transplantation. However, there are challenges with developing a stem cell differentiation protocol that can robustly generate mature insulin‐producing beta cells capable of glycemic control, as well as the manufacture and delivery of these cells at sufficient scales. It is clear that a significant trial and error requirement exists when establishing a manufacturing process to generate enough allogeneic cells for widespread use. Piecing together the current cell culture technology modalities to ensure cell product quality at scale is a complicated challenge that can include multiple decision thresholds during process design (eg, format of stem cell seed bank [vials or bags], adherent or suspension cell culture format with or without perfusion platforms, cell line [availability of equivalent clinical grade cells, male vs female sources], pluripotency state [naïve vs primed], passaging method [single cells vs clumps, enzymatic vs non‐enzymatic], passage number, extracellular matrix, growth media [fully defined or undefined], feeding strategy [eg, repeated batch, fed batch or continuous], type [continuous or intervals via cryopreservation stop‐point], length of production process [PSC expansion and directed differentiation], and scaled cell‐into‐device delivery systems).

**FIGURE 2:**
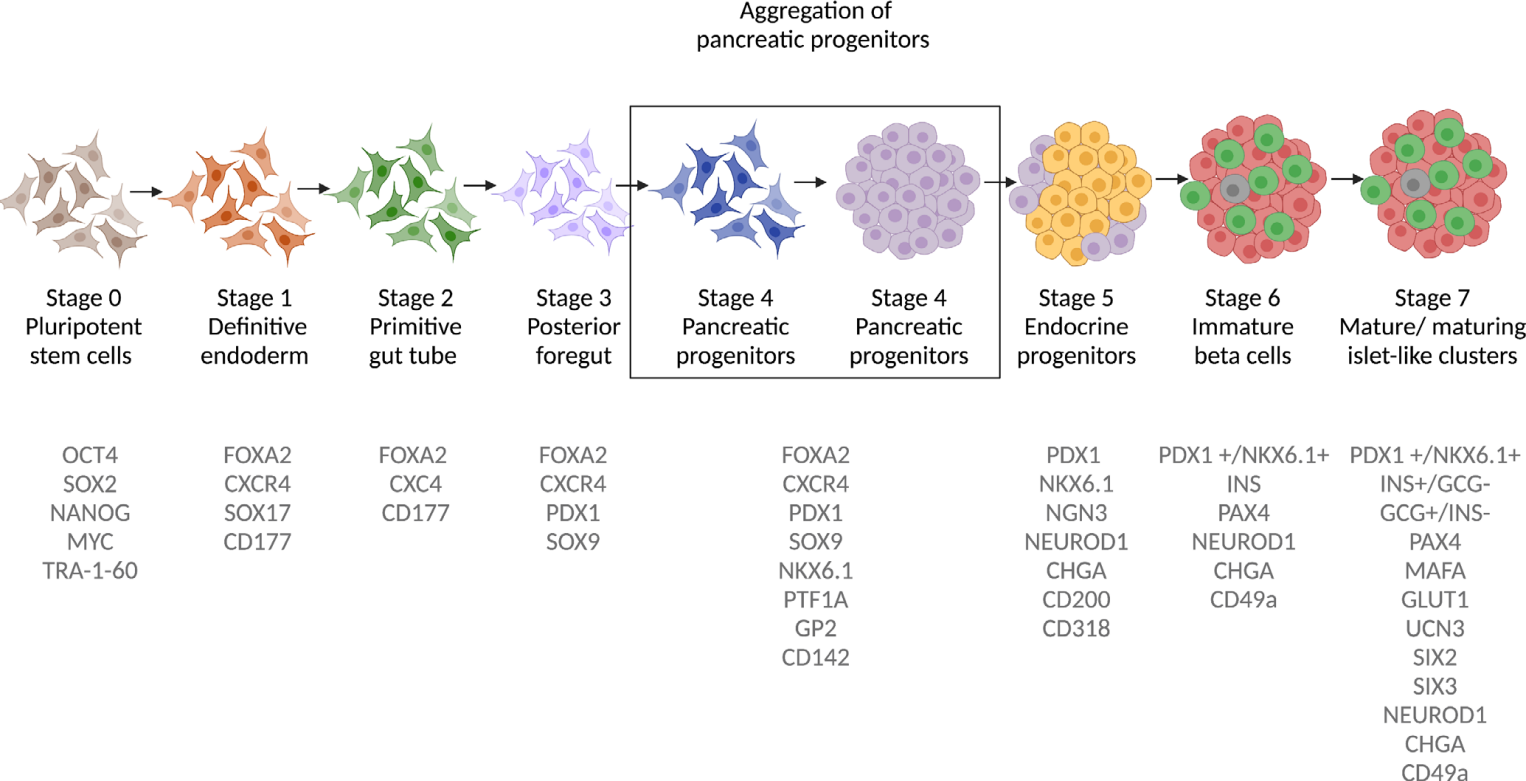
A representative schematic of the different stages during pluripotent stem cell (PSC) differentiation toward insulin‐producing cells. PSCs go through a seven‐stage protocol (adapted from Rezania et al^9^) using a combination of growth factors and small molecules. Each stage is identified by key proteins and transcription factors (depicted in gray text)

Several differentiation protocols use a stepwise multistage procedure to generate pancreatic progenitors and endocrine cells (Figure 2). D'Amour et al were the first to report human PSC‐derived pancreatic progenitors and immature hormone‐producing cells following the efficient induction of definitive endoderm using a combination of small molecules and growth factors to direct the differentiation.^10^, ^11^ Implant of these stem cell‐derived pancreatic progenitors and immature hormone‐producing cells into immunodeficient mice produced grafts with mature insulin‐producing beta cells that protected the mice against chemically induced hyperglycemia.^12^ Three phase 1/2 clinical trials led by ViaCyte Inc are currently active (ClinicalTrials.gov IDs: NCT04678557, NCT03163511, and NCT02239354) using product candidates PEC‐Encap and PEC‐Direct, that consist of a mix of human PSC‐derived pancreatic progenitor cells and immature hormone‐producing cells that are contained in retrievable macroencapsulation devices and implanted subcutaneously. The first device, PEC‐Encap, was designed to isolate and protect the graft from the immune cells while allowing insulin, glucose, oxygen, and waste products to diffuse through the device membranes. The initial clinical trial (ClinicalTrials.gov ID: NCT02239354) revealed that these cell‐containing devices were safe, free of off‐target cell growth, and protected against allo‐ and autoimmune rejection.^13^ However, unlike results observed in mouse studies,^12^ there was generally poor cell engraftment and survival, mostly likely due to hypoxia caused by a foreign body response which hampered vascularization of the cell‐containing device.^13^, ^14^ The subsequent clinical trial (ClinicalTrials.gov ID: NCT03163511) used a modified perforated encapsulation device that promotes vascularization of the graft and thus requires the use of immunosuppression by the recipients. Preliminary results from one patient implanted with the most current device configuration showed for the first time that implanted pancreatic human PSC‐derived progenitor cells survive, differentiate, and produce endogenous insulin as seen by clinically relevant increases in glucose‐responsive C‐peptide levels.^15^ Stimulated C‐peptide concentrations increased from 0.1 ng/mL (baseline) to 0.8 ng/mL post implant (week 39), associated with increased glycemic time in range (54%‐88%) and reduced average insulin use (39.5 units/day  at week 12 to 27.3 units/day at week 42). Results from these and subsequent trials will provide important information about the safety, durability during encapsulation, and efficacy of differentiated stem cells for the treatment of T1D in humans.

## 2 COMPARISON OF HUMAN PSC‐DERIVED PANCREATIC PROGENITORS AND ENDOCRINE CELLS FOR THE TREATMENT OF T1D

Pancreatic progenitors are a promising candidate as a therapeutic product for diabetes. These cells mature over months in vivo before becoming functional,^12^, ^16^, ^17^, ^18^ whereas PSC‐derived endocrine cells can reverse diabetes faster over a period of weeks.^9^ For patients who have lived many years with T1D, waiting for implanted PSC‐derived pancreatic progenitors to become functionally mature may not pose an additional burden if the ultimate outcome is an effective therapy that could minimize or eliminate the need for insulin injections and provide superior glycemic control and better quality of life. However, it has been reported that in rodents, the implantation site and encapsulation method used for PSC‐derived pancreatic progenitors can affect the proportion of endocrine cell types observed several months after implantation. For instance, Motté et al reported that pancreatic progenitors implanted in mice had a higher proportion of insulin‐producing cells when macroencapsulated in planar devices compared with those microencapsulated in alginate, which matured mostly into glucagon‐producing cells.^19^ Pepper et al showed that pancreatic progenitors implanted in a prevascularized subcutaneous site more effectively reversed hyperglycemia in mice in comparison to when the cells were implanted in the fat pad or non‐vascularized subcutaneous space.^20^ It has also been shown that the host sex, species (rats vs mice), and thyroid hormone levels can affect the rate of maturation and the acquisition of glucose competence in implanted pancreatic progenitor cells.^21^, ^22^, ^23^ The degree of microenvironment variability would likely be greater in the human population than it would in the lab setting using inbred strains of mice. As such, the host microenvironment, including levels of various circulating factors, could differ significantly in humans and might have an impact on the outcome of the pancreatic progenitor cell implants.

Despite potential caveats with implanting cells that are not terminally differentiated, pancreatic progenitors may offer significant advantages as a product compared with fully differentiated endocrine cells. For instance, pancreatic progenitors can be generated within a shorter culture time than mature islet‐like cells, potentially lowering cell production costs. A recent study showed that human PSC‐derived insulin‐producing cells and isolated human islets had a similar oxygen consumption rate (OCR) when challenged with high glucose.^24^ In contrast, PSC‐derived pancreatic progenitors have a lower OCR compared with human islets.^20^ These findings suggest that pancreatic progenitors may be relatively metabolically quiescent, a characteristic that could better support graft survival in a hypoxic environment. Cells are proliferative during the first four stages of differentiation to pancreatic progenitors^17^; however, cell losses typically occur during the later stages^9^ and will thereby contribute to the cost of cell manufacturing. Collectively, the time and cost to manufacture, cell yield, metabolic state, and the proven ability to effectively reverse hyperglycemia in rodent models make the use of PSC‐derived pancreatic progenitors appealing for the treatment of diabetes.

Over the last decade, significant progress has been made toward developing differentiation protocols that yield functionally mature insulin‐producing cells.^9^, ^12^, ^24^, ^25^, ^26^, ^27^, ^28^, ^29^ In some cases, PSC‐derived insulin‐producing cells generated entirely in vitro were capable of glucose‐stimulated insulin secretion^9^, ^24^, ^25^, ^26^, ^30^ as well as increased calcium signaling and mitochondrial respiration, similar to primary human islets.^24^ An implant of insulin‐producing cells can reverse diabetes in mice faster, and at a lower dose, than PSC‐derived pancreatic progenitors.^9^ More differentiated cell types may have a lower risk of outgrowth^9^, ^17^ following implant. Compared to pancreatic progenitors, cells further along in their differentiation may be less susceptible to becoming off‐target cell types resulting from uncontrolled environment cues in vivo. However, unlike for pancreatic progenitors, there is currently little reported on the effects of different implantation sites or encapsulation format on the graft function and composition when PSC‐derived insulin‐producing cells are used.

Many differentiation protocols take ~12 days to generate pancreatic progenitors compared to 28 to 61 days to make cell populations with a greater fraction of insulin‐producing cells.^9^, ^18^, ^24^, ^25^, ^26^, ^29^, ^31^ There are several efforts underway to reduce the time to generate mature insulin‐producing cells by overexpressing key transcription factors such as *PDX1*, *NKX6.1*, or *MAFA*. For example, a triple transfection of *Pdx1*, *Neurod1*, and *Mafa* in mouse iPSCs resulted in higher glucose‐stimulated insulin secretion after an 18‐day differentiation period compared to differentiated control cells that received empty vectors.^32^ These insulin‐producing cells reversed hyperglycemia in diabetic mice. Saxena et al used a synthetic lineage‐control network modulating the levels of the transcription factor *NGN3* (a master regulator of endocrine cell fate), *PDX1*, and *MAFA* during the last 11 days of a 24‐day differentiation protocol to make iPSC‐derived glucose responsive insulin‐producing cells.^33^ Using a 20‐day differentiation protocol, Zhu et al demonstrated that overexpressing *PDX1*, *NGN3*, and *MAFA* during stage 1 (definitive endoderm), stage 4 (pancreatic progenitors), and stage 6 (immature beta cells), respectively, resulted in glucose‐ and GLP‐1 responsive beta cells.^34^ Although presently there is no clinical data for the treatment of diabetes using implanted PSC‐derived insulin‐producing islet‐like clusters, results from preclinical studies have demonstrated their ability to reverse or protect against streptozotocin (STZ)‐induced hyperglycemia,^9^, ^24^, ^25^, ^26^, ^28^, ^29^ suggesting that they may be appropriate for cell replacement therapy. Vertex has recently announced a phase 1/2 clinical trial for VX‐880, a stem cell‐derived islet product, for the treatment of T1D in patients with hypoglycemia unawareness and severe hypoglycemia (ClinicalTrials.gov ID: NCT04786262).^35^ It remains to be established whether progenitor‐based or islet‐based stem cell‐derived cell products exhibit any relative enhancements in durability and efficacy toward the treatment of T1D.

## 3 A NEED FOR GREATER PROCESS UNDERSTANDING TO FACILITATE ROBUST, SCALED CELL PRODUCTION

The dose of donor pancreas cells typically infused during islet transplantation using the Edmonton protocol is 7000 to 12 000 islet equivalents (IEQ)/kg body weight.^6^, ^36^ Based on the estimated number of beta cells within an islet, this would translate into approximately a billion stem cell‐derived cells per recipient as a therapeutic dose, assuming equivalent survival and potency.^37^, ^38^ To facilitate manufacturing of cells at sufficient scale, adopting a scientific risk‐based analysis using a well‐structured quality‐by‐design strategy is beneficial.^39^ The maintenance of a cell product's critical quality attributes (CQAs), or physical, chemical, biological, or microbiological characteristics that the production process should control to be within appropriate limits, ensures the desired product quality required for therapeutic benefit. CQAs are controlled by the performance of the manufacturing process which drives their establishment. A control strategy during cell production is designed to monitor critical process parameters (CPPs), defined here as process criteria whose variability impacts CQAs.^39^, ^40^ Control of the various CPPs that impact the cells' CQAs should facilitate robust and consistent large‐scale production of a therapeutic stem cell‐derived cell product. Such CPPs could include a wide range of process inputs and outputs: parameters that influence process performance, material attributes that feed into the process, and defined user requirements for equipment utilized to facilitate the process (Table 1).

**TABLE 1:** An example of the quality‐by‐design process for the generation of pluripotent stem cell (PSC)‐derived insulin‐producing cells. The quality product profile outlines the properties of the desired clinical product based on the critical quality attributes (CQA). The critical process parameters are bioprocess parameters that influence the CQA

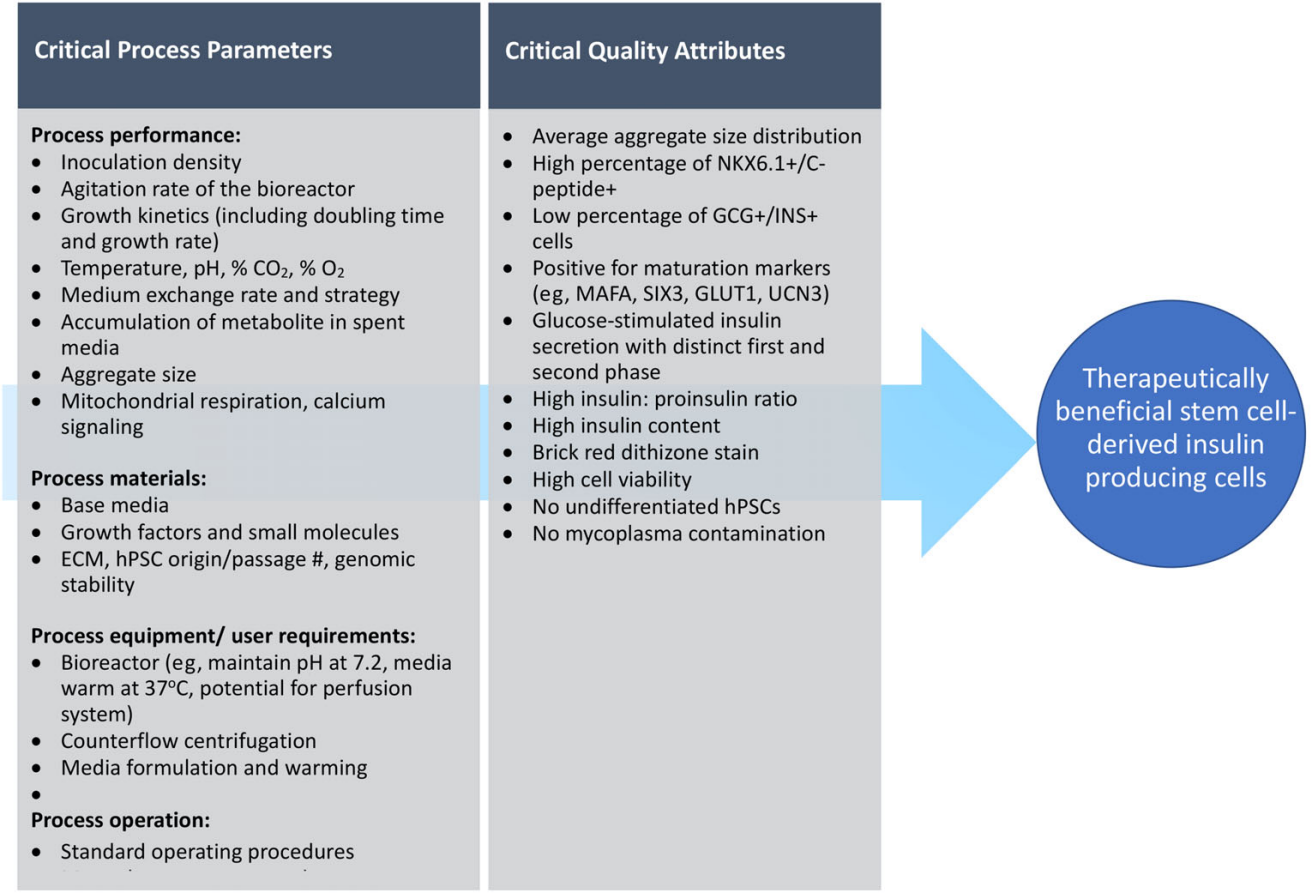

The decision to use either ESCs or iPSCs may be the first consideration when developing a large‐scale manufacturing plan. Although iPSCs were initially thought to be similar to ESCs based on morphology, growth rate, pluripotency markers, epigenetic status, and trilineage differentiation capability,^41^, ^42^ several studies observed differences in gene expression,^43^ DNA methylation,^44^ and persistent epigenetic memory,^45^ which may predispose the iPSCs to differentiate more efficiently to particular lineages. Some of the reported difference between ESCs and iPSCs may be due to the reprogramming procedure, incomplete reprogramming, as well as the method and duration of cell culture.^46^ Furthermore, Yamanaka noted that studies that reported differences between ESCs and iPSCs analyzed a relatively small number of clones (2‐12) compared to studies where no differences were observed (12‐68).^46^ The use of iPSCs may have less potential ethical and religious concerns associated with ESCs.^47^ Also, iPSCs may be superior for personalized cell therapies as there could be a lower risk of rejection and minimal need for immunosuppression upon implantation. In contrast, the transduction efficiency to generate iPSCs can be low, mutations arise during reprogramming, and accumulate as a result of prolonged passaging. Another consideration, albeit beyond the scope of this perspective, is picking the right cell line, irrespective of source (ESC vs iPSC) (reviewed by others^48^, ^49^, ^50^).

The method of passaging PSCs during routine maintenance and expansion may have an impact on the quality of the cells.^51^, ^52^, ^53^ Passaging may be done manually (using cell scrapers), mechanically (using a pipette to triturate the cells), using enzymes (eg, trypsin), or by non‐enzymatic dissociation (eg, using Versene). Furthermore, PSCs can be passaged as single cells or clumps depending on the downstream application. There are concerns that long‐term single‐cell passaging of PSCs may result in karyotype abnormalities,^54^, ^55^ but several groups have demonstrated normal karyotype after prolonged single‐cell passaging.^56^, ^57^, ^58^ For example, Cruvinel et al reported normal karyotype, sustained pluripotency, and trilineage differentiation capacity in human iPSCs after over 50 single‐cell passages.^57^ It is imperative that the karyotype of PSC banks is routinely monitored to ensure that chromosomal abnormalities do not accumulate over prolonged periods of passaging.

PSCs can be expanded either on a monolayer (a 2D process) or as aggregates in dynamic suspension cultures (a 3D process) to generate clinically relevant numbers of cells during routine maintenance of PSCs. Given the footprint required for monolayer expansion over several weeks, larger scale cell manufacturing might be challenging and expensive. Various cell culture platforms such as Cell Factory™, CellCube®, and HYPERFlask® vessels have been developed to provide a higher surface area for cell attachment and higher yield than conventional T‐flasks and multilayered CellSTACK® chambers. A major challenge with scalable 2D cell culture is the need to harvest the cells from the vessel surface. Large PSCs banks typically need to be generated, characterized for pluripotency and genetic stability, tested for potential contamination (eg, mycoplasma), and cryopreserved prior to the start of any downstream applications. It is imperative to develop strategies for a seed train that will allow for efficient culture and potential cryopreservation of both PSCs and their derivatives at the required scale. As the field of regenerative medicine expands, there is growing interest in developing bioprocesses that are performed in 3D dynamic suspension in order to address some of the challenges experienced with adherent monolayer culture, including high‐density culture via perfusion.^59^

**FIGURE 3:**
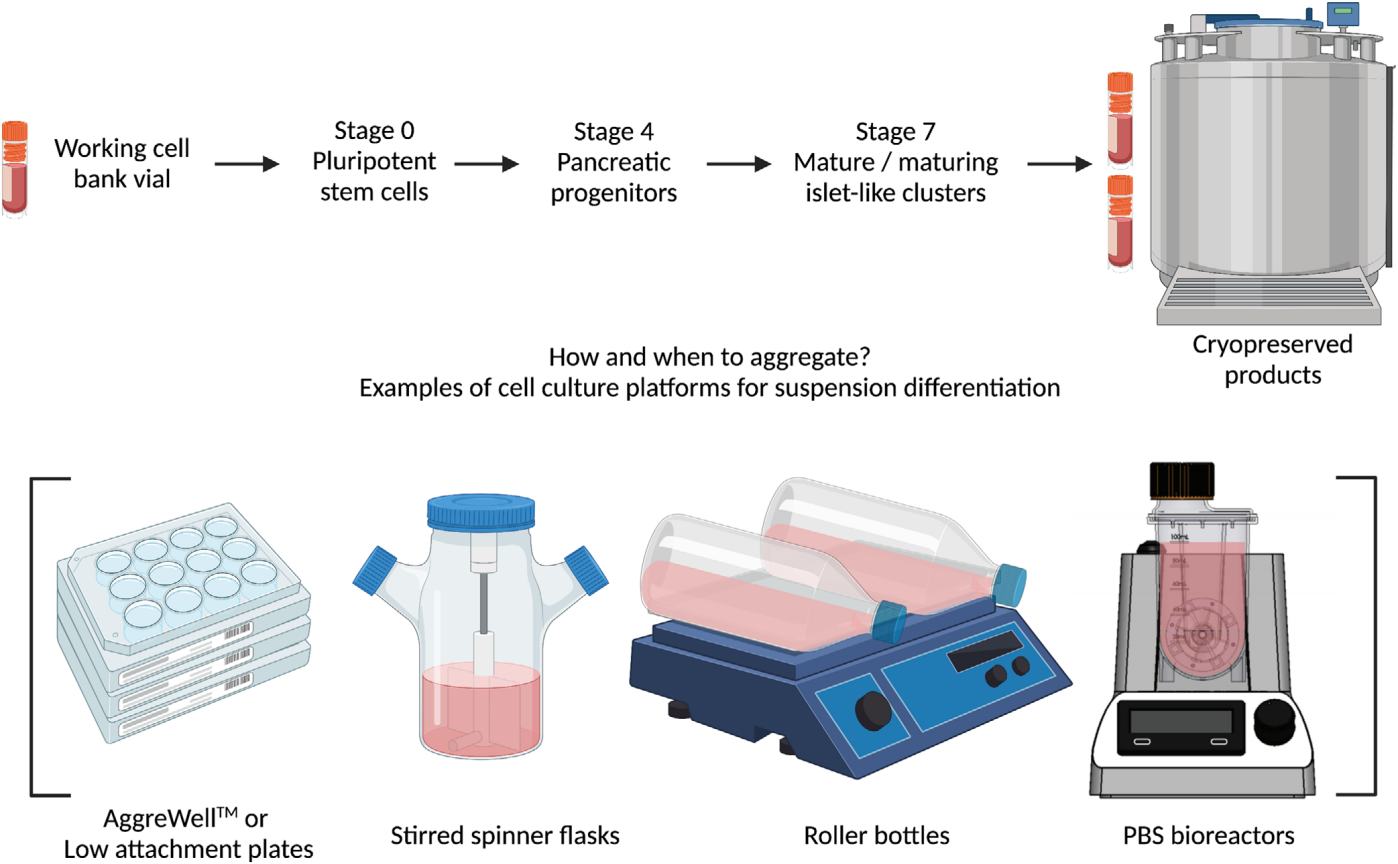
Schematic of sample bioprocessing strategies used for generating insulin‐producing cells from cryopreserved pluripotent stem cells (PSCs). Following the expansion of PSCs on a monolayer, the appropriate time point to generate cell aggregates still needs to be determined. Cell clusters may be generated using different available cell culture platforms such as low attachment plates, AggreWell plates, spinner flasks, roller bottles, or PBS bioreactors

Many differentiation protocols used to make insulin‐producing cells are initiated with 3D PSC aggregates (Figure 3).^18^, ^24^, ^25^, ^60^, ^61^ After generating enough cells, PSCs are typically harvested as single cells and seeded at a significantly higher density to initiate aggregate formation prior to the start of differentiation. There is also a short period between the initial high‐density seed of PSCs and the induction of the definitive endoderm stage, typically between 1 and 3 days. The generation of PSC aggregates is a common bottleneck to larger scale cell production because of the low efficiency of aggregate formation from single cells due to anoikis.^62^, ^63^ Significant cell losses have also been reported between 24 and 48 hours post aggregate formation.^62^, ^64^ The addition of Rho‐associated kinase (ROCK) inhibitors (eg, Y‐27632) has been shown to improve the survival of single cells.^62^, ^63^ Chen et al reported that a ROCK inhibitor (Chroman 1) supplemented with a pan‐caspase inhibitor (Emricasan), a mixture of polyamines, and a selective inhibitor of the integrated stress response (Trans‐ISRIB), improved cell survival approximately sixfold compared to cells treated with Y‐27632 alone.^65^

As aggregates grow over time, they become larger and can develop mass transport limitations of oxygen and nutrients that can lead to a necrotic cell core and reduced viability.^66^, ^67^ Aggregate formation can result in clusters with significant heterogeneity in their size distribution. This variability may result in differential morphogen effects between aggregates and negatively impact the differentiation efficiency. Borys et al demonstrated that PSC aggregates generated using a vertical wheeled bioreactor (PBS‐MINI) were more homogenously sized compared to those produced with a traditional horizontal blade bioreactor.^56^ PSC aggregate growth rate can be variable and slower compared to adherent cells,^53^ thus impacting the potential cell yield. The size and compaction of PSC aggregates affects the proliferative capacity of the clusters; smaller aggregates may have a slower unstable proliferative capacity, whereas larger aggregates may experience oxygen and nutrient deprivation resulting in poor viability.^68^, ^69^, ^70^, ^71^ Other challenges associated with 3D suspension cultures may include aggregate agglomeration which could result in necrotic cores due to hypoxia, and apoptosis due to the shear stress as a result of mixing. Several groups are actively working on improving the yield of PSC aggregates by improving bioreactor design, optimizing seeding density, feeding frequency, and the media formulation.^56^, ^72^, ^73^ Given the short time between seeding and initiating the differentiation, coupled with the low aggregate formation efficiencies and the slower growth of PSC aggregates, it is imperative that optimal process parameters be identified to minimize excessive material, labor, and financial costs associated with manufacturing large batches of cell clusters.

The formation of 3D aggregates appears to facilitate stem cell differentiation to endocrine lineages and promote regulated insulin secretion. For instance, generation of PSC‐derived pancreatic progenitor clusters increased gene expression of endocrine makers.^17^, ^74^ Furthermore, while pancreatic progenitors implanted in rodents as aggregates differentiated to insulin‐producing cells, similar non‐aggregated pancreatic progenitors failed to effectively differentiate into insulin‐producing cells within 16 weeks post‐transplant.^74^, ^75^ 3D aggregate architecture may be important for insulin secretion in primary islets and insulin‐secreting cell lines. Compared to purified single rat beta cells, both intact and reaggregated islets secreted four to five times more insulin in response to elevated glucose and leucine.^76^ These results were attributed to higher levels of cellular adenosine 3′,5′‐cyclic monophosphate (cAMP) in both intact and reaggregated islets in comparison to the single beta cells. Increased cAMP in beta cells promotes glucose‐competence and is a key downstream signaling molecule of glucagon and glucagon‐like peptide 1 (GLP‐1), both of which are produced by alpha cells.^77^, ^78^, ^79^ This could be why mixed aggregates containing alpha and beta cells are more glucose responsive than beta cell‐enriched aggregates.

Even aggregation of beta cells alone enhances glucose‐induced insulin secretion. For instance, aggregates generated from a mouse insulinoma cell line (MIN6), displayed better glucose‐stimulated insulin secretion in comparison with MIN6 cells grown on a monolayer.^80^ Purified rat beta cell aggregates implanted in diabetic mice could effectively reverse hyperglycemia, similar to intact islets, within 1 week, suggesting that the normal beta cell to non‐beta cell relationship may not be necessary for adequate glycemic control post‐transplant.^81^ However, the incorporation of non‐beta cells with reaggregated rat beta cells promoted long‐term survival of the implanted cells.^82^ It is noteworthy that Hogrebe et al achieved glucose‐responsiveness from PSC‐derived insulin‐producing cells in 2D planar culture through manipulation of the actin cytoskeleton, similar to that of cells made using a 3D suspension protocol.^26^ During the endocrine induction stage, depolymerization of filamentous actin using latrunculin A led to increased *NGN3* expression, and its downstream targets *NEUROD1* and *NKX2.2*, relative to that of untreated 2D controls at an equivalent time point of the differentiation. Furthermore, latrunculin A treated cells displayed glucose‐stimulated insulin secretion while the untreated 2D controls did not. Nevertheless, the cells differentiated in 2D were aggregated prior to implant into diabetic mice and resulted in the reversal of hyperglycemia more rapidly than cells generated using the 3D suspension protocol. Collectively, these studies highlight the impact of aggregate formation on nutrient and hormone‐mediated insulin secretion, and endocrine specification, based on the presence of key markers.

Aggregate size can have an impact on the survival and function of implanted cells.^24^, ^25^ Despite species differences in pancreas size, islet size is well conserved^83^ with an average of ~150 μm in diameter and a range of between 50 and 500 μm in mammals.^84^, ^85^ Lehmann et al demonstrated that islet size might play an important role in determining human islet transplantation outcome.^86^ In comparison to large islets (150‐300 μm diameter), smaller islets (50‐150 μm diameter) had a higher percentage of insulin‐positive cells per islet, higher insulin production, almost double the glucose‐stimulated insulin secretion based on perifusion assay, and less cell death following culture in hypoxic and normoxic conditions.^86^ Interestingly, following islet transplantation, two patients had equivalent stimulated C‐peptide levels despite the fact that one recipient received smaller islets and significantly less IEQ than the other that received larger islets (3352 IEQ/kg vs 11 625 IEQ/kg respectively).^86^ Previous reports have demonstrated that larger human and mouse islets secrete less insulin per IEQ compared to smaller islets.^87^, ^88^, ^89^, ^90^ The reaggregation of human islets has been employed to generate smaller and more homogeneously sized pseudoislets with improved post‐transplant survival and function.^91^, ^92^ These results indicate that the size of islets is an important variable affecting graft survival and function.

Similar reaggregation strategies used to generate pseudoislets have been adopted with stem cell‐derived insulin‐producing cells.^24^, ^25^ Velazco‐Cruz et al demonstrated that the reaggregation of clusters at the beginning of stage 6 (PSC‐derived beta cells), combined with the removal of ALK5i, a TFGβ inhibitor, resulted in the acquisition of glucose‐stimulated insulin secretion.^25^ Nair et al observed that reaggregation of PSC‐derived insulin‐producing cell clusters alone was not sufficient to induce glucose competence.^24^ In order for the cells to develop glucose‐stimulated insulin secretion, insulin positive cells were sorted prior to reaggregation and then cultured for an additional week.^24^ Several groups have used various sorting strategies to purify the cells based on different stage‐specific surface markers.^16^, ^60^, ^93^, ^94^ Pronounced cell losses associated with the dispersal, purification, and reaggregation of PSC‐derived clusters, although scarcely reported,^16^, ^60^ are expected. For instance, Kelly et al demonstrated that reaggregation of late stage clusters enriched the total endocrine population based on the percentage of chromogranin A, a pan‐endocrine marker.^16^ However, they cautioned that significant cell losses were associated with the reaggregation step. Stock et al observed progressive cell losses up to 8 days post reaggregation resulting in a yield of ~21%.^95^ Veres et al identified and used CD49a, a surface marker expressed on PSC‐derived beta cells,^60^ to purify cells and reported ~6% to 10% recovery. These reported yields suggest that current cell separation and enrichment techniques may be impractical for large‐scale cell manufacturing. Reporting the percentage of cell loss associated with enrichment and reaggregation is useful. Without such data, it is difficult to ascertain the impact these cell losses have on the overall yield of the target cell type(s) and the cost of cell production. It would be beneficial to determine whether the presumed cell losses occur due to non‐specific death or the negative selection of a suboptimal or off‐target population. One could mitigate the presumed cell losses by modifying the bioprocessing technique if it is due to non‐specific death (eg, addition of Y‐27632^16^), or by further optimizing the differentiation cocktail in order to generate a superior cell population based on stage‐specific protein levels, for example NKX6.1, and the acquisition of glucose‐stimulated insulin secretion.

An alternative bioprocessing strategy employed for the generation of PSC‐derived insulin‐producing cells involves differentiating cells on a monolayer toward the pancreatic progenitor stage before generating clusters using cell culture platforms such as spinner flasks, low attachment six‐well plates or air‐liquid interface (Figure 3).^9^, ^17^, ^75^ After that, the cell clusters may be further differentiated using static or dynamic 3D culture formats. Cells are most proliferative as they differentiate toward the pancreatic progenitor stage. As such, there may be an added advantage to having the cells attached as a monolayer during the first four stages of the differentiation instead of being aggregates. For instance, growth of aggregates formed during the early proliferative stages could result in potential mass transport limitations such that cells within the core may experience nutrient and oxygen deprivation. Poor diffusion of oxygen and nutrients in large aggregates could further result in decreased growth rate during these stages of differentiation. Such suboptimal parameters could result in lower cell yield of pancreatic progenitors while differentiating in aggregate form. Forming clusters when proliferation is minimal, typically after generating pancreatic progenitors, could allow better control of aggregate size and may obviate the need to reaggregate the clusters, a process that could result in additional cell loss during the last stage of the differentiation process.

## 4 DEMONSTRATION OF SCALABLE PRODUCTION OF HUMAN PSC‐DERIVED PANCREATIC PROGENITORS

In preparation for clinical trials, Schulz et al reported a human PSC expansion and differentiation workflow using their tightly controlled process parameters from 37 independent runs.^18^ These human PSC‐derived cell preparations containing pancreatic progenitor cells and immature hormone‐producing cells protected mice from STZ‐induced hyperglycemia after implantation.^18^ Following the thaw of high‐density cell banks containing 10^7^ CyT49 single cells/vial, the mean viability was 91.4% with a post‐thaw recovery of ~83%. Furthermore, the plating efficiency of these PSCs was ~80%. A single vial of cells had a cumulative fold expansion of about 270 within a 2‐week period, over four passages when cultured in standard T‐flasks. Differentiation of CyT49 cells was performed in suspension cultures using a 12‐day protocol.^18^ Prior to the start of each differentiation, an initial seeding density of 1 million single cells/mL was used to make PSC aggregates using low attachment six‐well plates rotating on an orbital shaker. CyT49 PSC aggregates had diameters between 100 and 200 μm and an aggregation efficiency (defined as the percentage of single cells incorporated into the cell clusters after one day) of ~75%. These results demonstrated improved recovery following single‐cell aggregation in comparison to other studies.^62^, ^64^ The quality of the pancreatic progenitors made between different runs and from separate vials of CyT49 PSCs was consistent based on the levels of key stage‐specific markers such as NKX6.1 and PDX1. On average, one stage 4 cell was generated per input PSC. The authors speculated that with the reported bioprocessing strategies, every vial containing 10^7^ CyT49 single cells could yield about 3.3 × 10^9^ pancreatic progenitors, estimated to be sufficient for about three patients. The bioprocessing strategies used in this study could be amendable to further scale‐up and automated production. A study modeling the cost of manufacturing PSC‐derived pancreatic progenitors estimated the range to be between ~CAD$55,000 and $300,000  per dose depending on the format of cell culture used (adherent vs suspension) and the number of doses produced per year (50 vs 500).^1^ While their study suggested that the most cost‐effective method to produce human PSC‐derived pancreatic progenitors could be achieved with high volume suspension cultures,^1^ the recommendation/pricing will change depending on ongoing breakthroughs of scale‐up technologies available. Similar bioprocess cost analyses have been performed by others.^96^, ^97^, ^98^ Bandeiras et al reported a comprehensive bioprocessing cost model for manufacturing beta cells from PSCs taking into account numerous variables such as the number of batches produced per year, the number of patients that could be implanted from cells generated per batch, and the number of patients that could implanted with the cells per year.^96^ Based on their model, the projected cost of manufacturing beta cells for one patient could be as high as $427,231. Their simulation showed that the majority of the production cost would be spent on reagents used during the differentiation period. Furthermore, they speculated that the cost of goods could be reduced by optimizing downstream processing to improve cell yield. While such modeling studies provide invaluable information on the economic implications of cell therapy for the treatment of T1D, the true cost of manufacturing PSC‐derived pancreatic progenitors and endocrine cells is yet to be determined and undoubtedly efficiencies will be improved upon.

## 5 CONCLUSION

Although islet transplantation demonstrates proof‐of‐concept for cell replacement therapy to treat T1D, the widespread implementation of this procedure is limited by paucity of donor islets. The potential of stem cell‐derived insulin‐producing cells for the treatment of diabetes has been demonstrated using rodent models. Furthermore, the safety and efficacy of PSC‐derived pancreatic progenitors is currently being evaluated in ongoing clinical trials. Differentiation protocols are being developed using multiple cell lines. It is desirable to identify critical parameters that will affect the CQAs of the target product profile. Adopting quality‐by‐design methodology can help unify the work being done across the field and accelerate the efforts toward successful clinical translation, large‐scale cell manufacturing and commercialization.

## CONFLICT OF INTEREST

S.R. is an employee and a stock/shareholder of ViaCyte, Inc. Relevant to the topic of this manuscript, T.J.K. is an inventor on patents and has done consulting for, and/or received research support from, ViaCyte Inc, Sigilon Therapeutics, CRISPR Therapeutics, and STEMCELL Technologies. Diepiriye G. Iworima indicated no potential conflicts of interest.

## Data Availability

Data sharing is not applicable to this article as no new data were created or analyzed in this study.

## References

[1] Wallner K , Pedroza RG , Awotwe I, et al. Stem cells and beta cell replacement therapy: a prospective health technology assessment study. BMC Endocr Disord. 2018;18:1‐12. 29382312 10.1186/s12902-018-0233-7 PMC5791348

[2] Balakumar P , Maung‐U K , Jagadeesh G . Prevalence and prevention of cardiovascular disease and diabetes mellitus. Pharmacol Res. 2016;113:600‐609. 27697647 10.1016/j.phrs.2016.09.040

[3] IDF . IDF Diabetes Atlas 9th edition 2019; 2019. https://www.diabetesatlas.org/en/. Accessed October 21, 2020.

[4] Bommer C , Sagalova V , Heesemann E , et al. Global economic burden of diabetes in adults: projections from 2015 to 2030. Diabetes Care. 2018;41:963‐970. 29475843 10.2337/dc17-1962

[5] Bliss M . Banting's, Best's and Collip's accounts of the discovery of insulin. Bull Hist Med. 1982;56:554‐568. 6760943

[6] Shapiro AMJ , Lakey JRT , Ryan EA , et al. Islet transplantation in seven patients with type 1 diabetes mellitus using a glucocorticoid‐free immunosuppressive regimen. N Engl J Med. 2000;343:230‐238. 10911004 10.1056/NEJM200007273430401

[7] Andres A , Kin T , O'Gorman D , et al. Clinical islet isolation and transplantation outcomes with deceased cardiac death donors are similar to neurological determination of death donors. Transpl Int. 2016;29:34‐40. 26264982 10.1111/tri.12650

[8] Markmann JF , Deng S , Desai NM , et al. The use of non‐heart‐beating donors for isolated pancreatic islet transplantation. Transplantation. 2003;75:1423‐1429. 12792491 10.1097/01.TP.0000061119.32575.F4

[9] Rezania A , Bruin JE , Arora P , et al. Reversal of diabetes with insulin‐producing cells derived in vitro from human pluripotent stem cells. Nat Biotechnol. 2014;32:1121‐1133. 25211370 10.1038/nbt.3033

[10] D'Amour KA , Bang AG , Eliazer S , et al. Production of pancreatic hormone‐expressing endocrine cells from human embryonic stem cells. Nat Biotechnol. 2006;24:1392‐1401. 17053790 10.1038/nbt1259

[11] D'Amour KA , Agulnick AD , Eliazer S , Kelly OG , Kroon E , Baetge EE . Efficient differentiation of human embryonic stem cells to definitive endoderm. Nat Biotechnol. 2005;23:1534‐1541. 16258519 10.1038/nbt1163

[12] Kroon E , Martinson LA , Kadoya K , et al. Pancreatic endoderm derived from human embryonic stem cells generates glucose‐responsive insulin‐secreting cells in vivo. Nat Biotechnol. 2008;26:443‐452. 18288110 10.1038/nbt1393

[13] Henry RR et al. Initial clinical evaluation of VC‐01TM combination product—a stem cell–derived islet replacement for type 1 diabetes (T1D). Diabetes. 2018;67:138‐OR.

[14] Pullen L , Stem C . Cell‐derived pancreatic progenitor cells have now been transplanted into patients: report from IPITA 2018. Am J Transplant. 2018;18:1581‐1582. 29979835 10.1111/ajt.14954

[15] Keymeulen B et al. 196‐LB: stem cell‐derived islet replacement therapy (VC‐02) demonstrates production of C‐peptide in patients with type 1 diabetes (T1D) and hypoglycemia unawareness. Diabetes. 2021;70:196‐LB.33055188

[16] Kelly OG , Chan MY , Martinson LA , et al. Cell‐surface markers for the isolation of pancreatic cell types derived from human embryonic stem cells. Nat Biotechnol. 2011;29:750‐756. 21804561 10.1038/nbt.1931

[17] Rezania A , Bruin JE , Riedel MJ , et al. Maturation of human embryonic stem cell‐derived pancreatic progenitors into functional islets capable of treating pre‐existing diabetes in mice. Diabetes. 2012;61:2016‐2029. 22740171 10.2337/db11-1711 PMC3402300

[18] Schulz TC , Young HY , Agulnick AD , et al. A scalable system for production of functional pancreatic progenitors from human embryonic stem cells. PLoS One. 2012;7:e37004. 22623968 10.1371/journal.pone.0037004 PMC3356395

[19] Motté E et al. Composition and function of macroencapsulated human embryonic stem cell‐derived implants: comparison with clinical human islet cell grafts. Am J Physiol Metab. 2014;307:E838‐E846. 10.1152/ajpendo.00219.2014 25205822

[20] Pepper AR , Pawlick R , Bruni A , et al. Transplantation of human pancreatic endoderm cells reverses diabetes post transplantation in a prevascularized subcutaneous site. Stem Cell Rep. 2017;8:1689‐1700. 10.1016/j.stemcr.2017.05.004 PMC5470173 28591651

[21] Saber N , Bruin JE , O'Dwyer S , Schuster H , Rezania A , Kieffer TJ . Sex differences in maturation of human embryonic stem cell–derived β cells in mice. Endocrinology. 2018;159:1827‐1841. 29420708 10.1210/en.2018-00048

[22] Bruin JE , Saber N , O'Dwyer S , et al. Hypothyroidism impairs human stem cell‐derived pancreatic progenitor cell maturation in mice. Diabetes. 2016;65:1297‐1309. 26740603 10.2337/db15-1439

[23] Bruin JE , Asadi A , Fox JK , Erener S , Rezania A , Kieffer TJ . Accelerated maturation of human stem cell‐derived pancreatic progenitor cells into insulin‐secreting cells in immunodeficient rats relative to mice. Stem Cell Rep. 2015;5:1081‐1096. 10.1016/j.stemcr.2015.10.013 PMC4682152 26677767

[24] Nair GG et al. Recapitulating endocrine cell clustering in culture promotes maturation of human stem‐cell‐derived β cells. Nat Cell Biol. 2019;21:263. 30710150 10.1038/s41556-018-0271-4 PMC6746427

[25] Velazco‐Cruz L , Song J , Maxwell KG , et al. Acquisition of dynamic function in human stem cell‐derived β cells. Stem Cell Rep. 2019;12:351‐365. 10.1016/J.STEMCR.2018.12.012 PMC6372986 30661993

[26] Hogrebe NJ , Augsornworawat P , Maxwell KG , Velazco‐Cruz L , Millman JR . Targeting the cytoskeleton to direct pancreatic differentiation of human pluripotent stem cells. Nat Biotechnol. 2020;38(4):460‐470. 10.1038/s41587-020-0430-6 32094658 PMC7274216

[27] Millman JR , Xie C , van Dervort A , Gürtler M , Pagliuca FW , Melton DA . Generation of stem cell‐derived β‐cells from patients with type 1 diabetes. Nat Commun. 2016;7:11463. 27163171 10.1038/ncomms11463 PMC4866045

[28] Maxwell KG et al. Gene‐edited human stem cell–derived β cells from a patient with monogenic diabetes reverse preexisting diabetes in mice. Sci Transl Med. 2020;12:eaax9106. 32321868 10.1126/scitranslmed.aax9106 PMC7233417

[29] Pagliuca FW , Millman JR , Gürtler M , et al. Generation of functional human pancreatic β cells In vitro. Cell. 2014;159:428‐439. 25303535 10.1016/j.cell.2014.09.040 PMC4617632

[30] Hrvatin S , O'Donnell CW , Deng F , et al. Differentiated human stem cells resemble fetal, not adult, β cells. Proc Natl Acad Sci USA. 2014;111:3038‐3043. 10.1073/pnas.1400709111 24516164 PMC3939927

[31] Mahaddalkar PU , Scheibner K , Pfluger S , et al. Generation of pancreatic β cells from CD177 + anterior definitive endoderm. Cell Biol. 2020;38:1061‐1072. 10.1038/s41587-020-0492-5 32341565

[32] Wang L , Huang Y , Guo Q , et al. Differentiation of iPSCs into insulin‐producing cells via adenoviral transfection of PDX‐1, NeuroD1 and MafA. Diabetes Res Clin Pract. 2014;104:383‐392. 24794627 10.1016/j.diabres.2014.03.017

[33] Saxena P et al. A programmable synthetic lineage‐control network that differentiates human IPSCs into glucose‐sensitive insulin‐secreting beta‐like cells. Nat Commun. 2016;7:1‐14. 10.1038/ncomms11247 PMC4831023 27063289

[34] Zhu Y , Tonne JM , Liu Q , et al. Targeted derivation of organotypic glucose‐ and GLP‐1‐responsive β cells prior to transplantation into diabetic recipients. Stem Cell Rep. 2019;13:307‐321. 10.1016/j.stemcr.2019.07.006 PMC6700523 31378674

[35] Vertex Announces FDA . Clearance of investigational new drug (IND) application for VX‐880, a novel cell therapy for the treatment of Type 1 diabetes (T1D) | Vertex Newsroom. https://news.vrtx.com/press-release/vertex-announces-fda-clearance-investigational-new-drug-ind-application-vx-880-novel. Accessed February 7, 2021.

[36] Shapiro AMJ , Pokrywczynska M , Ricordi C . Clinical pancreatic islet transplantation. Nat Rev Endocrinol. 2017;13:268‐277. 27834384 10.1038/nrendo.2016.178

[37] Docherty K , Bernardo AS , Vallier L . Embryonic stem cell therapy for diabetes mellitus. Semin Cell Dev Biol. 2007;18:827‐838. 17959396 10.1016/j.semcdb.2007.09.009

[38] Lock LT , Tzanakakis ES . Stem/progenitor cell sources of insulin‐producing cells for the treatment of diabetes. Tissue Eng. 2007;13:1399‐1412. 17550339 10.1089/ten.2007.0047

[39] Lipsitz YY , Timmins NE , Zandstra PW . Quality cell therapy manufacturing by design. Nat Biotechnol. 2016;34:393‐400. 27054995 10.1038/nbt.3525

[40] Witcher M. Why controlling CQAs isn't good enough for gene cell therapies; 2020. https://www.cellandgene.com/doc/why‐controlling‐cqas‐isn‐t‐good‐enough‐for‐gene‐cell‐therapies‐0001?vm_tId=2205493&user=6865a237‐0418‐4d2d‐b925‐6c6870a56ce6&vm_alias=Why Controlling CQAs Isn%27t Good Enough For Cell And%26nbsp;Gene Therapies&utm_source=mk. Accessed March 20, 2021.

[41] Takahashi K , Tanabe K , Ohnuki M , et al. Induction of pluripotent stem cells from adult human fibroblasts by defined factors. Cell. 2007;131:861‐872. 18035408 10.1016/j.cell.2007.11.019

[42] Yu J et al. Induced pluripotent stem cell lines derived from human somatic cells. Science (80‐). 2007;318:1917‐1920. 10.1126/science.1151526 18029452

[43] Chin MH , Mason MJ , Xie W , et al. Induced pluripotent stem cells and embryonic stem cells are distinguished by gene expression signatures. Cell Stem Cell. 2009;5:111‐123. 19570518 10.1016/j.stem.2009.06.008 PMC3448781

[44] Deng J , Shoemaker R , Xie B , et al. Targeted bisulfite sequencing reveals changes in DNA methylation associated with nuclear reprogramming. Nat Biotechnol. 2009;27:353‐360. 19330000 10.1038/nbt.1530 PMC2715272

[45] Kim K , Zhao R , Doi A , et al. Donor cell type can influence the epigenome and differentiation potential of human induced pluripotent stem cells. Nat Biotechnol. 2011;29:1117‐1119. 22119740 10.1038/nbt.2052 PMC3357310

[46] Yamanaka S . Induced pluripotent stem cells: past, present, and future. Cell Stem Cell. 2012;10:678‐684. 22704507 10.1016/j.stem.2012.05.005

[47] Moradi S et al. Research and therapy with induced pluripotent stem cells (iPSCs): social, legal, and ethical considerations. Stem Cell Res Ther. 2019;10:1‐13. 31753034 10.1186/s13287-019-1455-y PMC6873767

[48] Stevens KR , Murry CE . Human pluripotent stem cell‐derived engineered tissues: clinical considerations. Cell Stem Cell. 2018;22:294‐297. 29499147 10.1016/j.stem.2018.01.015 PMC6344123

[49] Stacey GN , Andrews PW , Barbaric I , et al. Stem cell culture conditions and stability: a joint workshop of the PluriMes Consortium and Pluripotent Stem Cell Platform. Regen Med. 2019;14:243‐255. 30938271 10.2217/rme-2019-0001 PMC7611410

[50] Why do scientists need many lines of embryonic stem cells? Nat Rep Stem Cells 2007. doi:10.1038/stemcells.2007.18

[51] Beers J , Gulbranson DR , George N , et al. Passaging and colony expansion of human pluripotent stem cells by enzyme‐free dissociation in chemically defined culture conditions. Nat Protoc. 2012;7:2029‐2040. 23099485 10.1038/nprot.2012.130 PMC3571618

[52] Zakrzewski W , Dobrzyński M , Szymonowicz M , Rybak Z . Stem cells: past, present, and future. Stem Cell Res Ther. 2019;10:1‐22. 30808416 10.1186/s13287-019-1165-5 PMC6390367

[53] Chen KG , Mallon BS , McKay RDG , Robey PG . Human pluripotent stem cell culture: considerations for maintenance, expansion, and therapeutics. Cell Stem Cell. 2014;14:13‐26. 24388173 10.1016/j.stem.2013.12.005 PMC3915741

[54] Garitaonandia I , Amir H , Boscolo FS , et al. Increased risk of genetic and epigenetic instability in human embryonic stem cells associated with specific culture conditions. PLoS One. 2015;10:e0118307. 25714340 10.1371/journal.pone.0118307 PMC4340884

[55] Bai Q , Ramirez JM , Becker F , et al. Temporal analysis of genome alterations induced by single‐cell passaging in human embryonic stem cells. Stem Cells Dev. 2015;24:653‐662. 25254421 10.1089/scd.2014.0292 PMC4333508

[56] Borys BS , Dang T , So T , et al. Overcoming bioprocess bottlenecks in the large‐scale expansion of high‐quality hiPSC aggregates in vertical‐wheel stirred suspension bioreactors. Stem Cell Res Ther. 2021;12:55. 33436078 10.1186/s13287-020-02109-4 PMC7805206

[57] Cruvinel E , Ogusuku I , Cerioni R , et al. Long‐term single‐cell passaging of human iPSC fully supports pluripotency and high‐efficient trilineage differentiation capacity. SAGE open Med. 2020;8:2050312120966456. 33149912 10.1177/2050312120966456 PMC7586033

[58] Thomson A , Wojtacha D , Hewitt Z , et al. Human embryonic stem cells passaged using enzymatic methods retain a normal karyotype and express CD30. Cloning Stem Cells. 2008;10:89‐105. 18241127 10.1089/clo.2007.0072

[59] Manstein F et al. High density bioprocessing of human pluripotent stem cells by metabolic control and in silico modeling. Stem Cells Translational Medicine. 2021;10:1063‐1080. 10.1002/sctm.20-0453 33660952 PMC8235132

[60] Veres A , Faust AL , Bushnell HL , et al. Charting cellular identity during human in vitro β‐cell differentiation. Nat. 2019;2019(1):368‐373. 10.1038/s41586-019-1168-5 PMC6903417 31068696

[61] Sahabian A , Dahlmann J , Martin U . Production and cryopreservation of definitive endoderm from human pluripotent stem cells under defined and scalable culture conditions. Nat Protoc. 2021;16:1581‐1599. 10.1038/s41596-020-00470-5 33580232

[62] Watanabe K , Ueno M , Kamiya D , et al. A ROCK inhibitor permits survival of dissociated human embryonic stem cells. Nat Biotechnol. 2007;25:681‐686. 17529971 10.1038/nbt1310

[63] Chen G , Hou Z , Gulbranson DR , Thomson JA . Actin‐myosin contractility is responsible for thcelle reduced viability of dissociated human embryonic stem cells. Cell Stem Cell. 2010;7:240‐248. 20682449 10.1016/j.stem.2010.06.017 PMC2916864

[64] Lipsitz YY , Tonge PD , Zandstra PW . Chemically controlled aggregation of pluripotent stem cells. Biotechnol Bioeng. 2018;115:2061‐2066. 29679475 10.1002/bit.26719 PMC6055717

[65] Chen Y , Tristan CA , Chen L , et al. A versatile polypharmacology platform promotes cytoprotection and viability of human pluripotent and differentiated cells. Nat Methods. 2021;18:528‐541. 33941937 10.1038/s41592-021-01126-2 PMC8314867

[66] Komatsu H , Cook C , Wang CH , et al. Oxygen environment and islet size are the primary limiting factors of isolated pancreatic islet survival. PLoS One. 2017;12(8):e0183780. 10.1371/journal.pone.0183780. 28832685 PMC5568442

[67] Sachlos E , Auguste DT . Embryoid body morphology influences diffusive transport of inductive biochemicals: a strategy for stem cell differentiation. Biomaterials. 2008;29:4471‐4480. 18793799 10.1016/j.biomaterials.2008.08.012

[68] Nath SC , Horie M , Nagamori E , Kino‐oka M . Size‐ and time‐dependent growth properties of human induced pluripotent stem cells in the culture of single aggregate. J Biosci Bioeng. 2017;124:469‐475. 28601606 10.1016/j.jbiosc.2017.05.006

[69] Kato Y , Kim M‐H , Kino‐oka M . Comparison of growth kinetics between static and dynamic cultures of human induced pluripotent stem cells. J Biosci Bioeng. 2018;125:736‐740. 29398548 10.1016/j.jbiosc.2018.01.002

[70] Kim MH , Kino‐oka M . A novel strategy for simple and robust expansion of human pluripotent stem cells using botulinum hemagglutinin. Adv Exper Med and Bio. Vol 1077. Singapore: Springer; 2018:19‐29. 30357681 10.1007/978-981-13-0947-2_2

[71] Kim MH , Takeuchi K , Kino‐oka M . Role of cell‐secreted extracellular matrix formation in aggregate formation and stability of human induced pluripotent stem cells in suspension culture. J Biosci Bioeng. 2018;127:372‐380. 30249415 10.1016/j.jbiosc.2018.08.010

[72] Kropp C , Massai D , Zweigerdt R . Progress and challenges in large‐scale expansion of human pluripotent stem cells. Process Biochem. 2017;59:244‐254.

[73] Nogueira DES et al. Strategies for the expansion of human induced pluripotent stem cells as aggregates in single‐use Vertical‐Wheel™ bioreactors. J Biol Eng. 2019;13(1):1‐14. 31534477 10.1186/s13036-019-0204-1 PMC6744632

[74] Bruin JE , Rezania A , Xu J , et al. Maturation and function of human embryonic stem cell‐derived pancreatic progenitors in macroencapsulation devices following transplant into mice. Diabetologia. 2013;56:1987‐1998. 10.1007/s00125-013-2955-4 23771205

[75] Toyoda T , Mae SI , Tanaka H , et al. Cell aggregation optimizes the differentiation of human ESCs and iPSCs into pancreatic bud‐like progenitor cells. Stem Cell Res. 2015;14:185‐197. 25665923 10.1016/j.scr.2015.01.007

[76] Pipeleers DG et al. Interplay of nutrients and hormones in the regulation of insulin release. Endocrinology. 1985;117:824‐833. 2862021 10.1210/endo-117-3-824

[77] Holz GG IV , Kiihtreiber WM , Habener JF . Pancreatic beta‐cells are rendered glucose‐competent by the insulinotropic hormone glucagon‐like peptide‐1(7‐37). Nature. 1993;361:362‐365. 8381211 10.1038/361362a0 PMC2916679

[78] Capozzi, ME , Svendsen B , Encisco SE *et al*. β‐Cell tone is defined by proglucagon peptides through cyclic AMP signaling JCI Insight. 2019;4(5):e126742. 10.1172/jci.insight.126742 4 PMC6483521 30720465

[79] Svendsen B , Larsen O , Gabe MBN , Christiansen CB , Rosenkilde MM , Drucker DJ , Holst JJ . Insulin secretion depends on intra‐islet glucagon signaling. Cell Rep. 2018;25(5):1127‐1134. 10.1016/j.celrep.2018.10.018 30380405

[80] Chowdhury A , Dyachok O , Tengholm A , Sandler S , Bergsten P . Functional differences between aggregated and dispersed insulin‐producing cells. Diabetologia. 2013;56:1557‐1568. 23604550 10.1007/s00125-013-2903-3 PMC3671110

[81] King AJF , Fernandes JR , Hollister‐Lock J , Nienaber CE , Bonner‐Weir S , Weir GC . Normal relationship of beta‐ and non‐beta‐cells not needed for successful islet transplantation. Diabetes. 2007;56:2312‐2318. 17563059 10.2337/db07-0191

[82] Pipeleers DG , Pipeleers‐Marichal M , Hannaert JC , et al. Transplantation of purified islet cells in diabetic rats: I. Standardization of islet cell grafts. Diabetes. 1991;40:908‐919. 2060727 10.2337/diab.40.7.908

[83] Kieffer TJ . Closing in on mass production of mature human beta cells. Cell Stem Cell. 2016;18:699‐702. 27257758 10.1016/j.stem.2016.05.014

[84] Ionescu‐Tirgoviste C , Gagniuc PA , Gubceac E , et al. A 3D map of the islet routes throughout the healthy human pancreas. Sci Rep. 2015;5:14634. 26417671 10.1038/srep14634 PMC4586491

[85] Kim A , Miller K , Jo J , Kilimnik G , Wojcik P , Hara M . Islet architecture: a comparative study. Islets. 2009;1:129‐136. 20606719 10.4161/isl.1.2.9480 PMC2894473

[86] Lehmann R , Zuellig RA , Kugelmeier P , et al. Superiority of small islets in human islet transplantation. Diabetes. 2007;56:594‐603. 17327426 10.2337/db06-0779

[87] Macgregor RR et al. Small rat islets are superior to large islets in in vitro function and in transplantation outcomes Rat Islet Isolation and Separation Procedures. Am J Physiol Endocrinol Metab. 2006;290:771‐779. 10.1152/ajpendo.00097.2005 16303846

[88] Fujita Y , Takita M , Shimoda M , et al. Large human islets secrete less insulin per islet equivalent than smaller islets in vitro. Islets. 2011;3:1‐5. 21266855 10.4161/isl.3.1.14131

[89] Su Z , Xia J , Shao W , et al. Small islets are essential for successful intraportal transplantation in a diabetes mouse model. Scand J Immunol. 2010;72:504‐510. 21044124 10.1111/j.1365-3083.2010.02466.x

[90] Farhat B , Almelkar A , Ramachandran K , et al. Small human islets comprised of more β‐cells with higher insulin content than large islets. Islets. 2013;5:87‐94. 23648896 10.4161/isl.24780 PMC4204020

[91] Yu Y , Gamble A , Pawlick R , et al. Bioengineered human pseudoislets form efficiently from donated tissue, compare favourably with native islets in vitro and restore normoglycaemia in mice. Diabetologia. 2018;61:2016‐2029. 29971529 10.1007/s00125-018-4672-5 PMC6096633

[92] Ichihara Y , Utoh R , Yamada M , Shimizu T , Uchigata Y . Size effect of engineered islets prepared using microfabricated wells on islet cell function and arrangement. Heliyon. 2016;2:e00129. 27441299 10.1016/j.heliyon.2016.e00129 PMC4946309

[93] Cogger KF , Sinha A , Sarangi F , et al. Glycoprotein 2 is a specific cell surface marker of human pancreatic progenitors. Nat Commun. 2017;8:331. 28835709 10.1038/s41467-017-00561-0 PMC5569081

[94] Ameri J , Borup R , Prawiro C , et al. Efficient generation of glucose‐responsive beta cells from isolated GP2+ human pancreatic progenitors. Cell Rep. 2017;19:36‐49. 28380361 10.1016/j.celrep.2017.03.032

[95] Stock AA , Manzoli V , de Toni T , et al. Conformal coating of stem cell‐derived islets for β cell replacement in type 1 diabetes. Stem Cell Rep. 2019;14:91‐104. 10.1016/j.stemcr.2019.11.004 PMC6962554 31839542

[96] Bandeiras C , Cabral JMS , Gabbay RA , Finkelstein SN , Ferreira FC . Bringing stem cell‐based therapies for type 1 diabetes to the clinic: early insights from bioprocess economics and cost‐effectiveness analysis. Biotechnol J. 2019;14:1800563. 10.1002/biot.201800563 31127682

[97] Archibald PRT , Williams DJ . Using the cost‐effectiveness of allogeneic islet transplantation to inform induced pluripotent stem cell‐derived β‐cell therapy reimbursement. Regen Med. 2015;10:959‐973. 26563421 10.2217/rme.15.59

[98] Bandeiras C et al. Economics of beta‐cell replacement therapy. Curr Diab Rep. 2019;19:1‐8. 31375935 10.1007/s11892-019-1203-9

